# Analyzing the epidemic situation and influencing factors of childhood pneumonia during the COVID-19 epidemic period in Laiwu, China

**DOI:** 10.1097/MD.0000000000035052

**Published:** 2023-09-01

**Authors:** Jimei Yang, Junqing Li, Ruiliang Lv, Xiang Wang, Chunfeng Zheng, Yan Gu, Mingzhu Zhang

**Affiliations:** a Jinan Second Maternal and Child Health Hospital, Jinan, Shandong, China; b Affiliated Hospital of Qingdao University, Jinan, Shandong, China

**Keywords:** childhood pneumonia, COVID-19, epidemiology, imaging diagnosis, influencing factors, respiratory infections

## Abstract

This study aimed to analyze the trends of childhood pneumonia before and after the corona virus disease 2019 (COVID-19) pandemic in Laiwu, China, and explore the associated risk factors to provide a reference for the prevention, control, and treatment of childhood pneumonia. In this cross-sectional study, data were collected from 11,716 children aged 3 to 12 years who underwent chest radiography/computed tomography from January 2018 to December 2021 in Laiwu, China. The generalized estimating equation model was used to analyze the time trend of the pneumonia detection rate. Multivariate logistic regression analysis was used to analyze the risk factors for pneumonia in children. The overall pneumonia detection rate was 40.3% (4721/11,716). The pneumonia detection rate was 41.5% and 39.5% in 2018 and 2019, respectively, before the COVID-19 pandemic, which decreased to 38.1% in 2020 during the pandemic and increased to 40.7% in 2021 after the pandemic. Multivariate logistic regression analysis showed that preterm birth (odds ratio [OR] = 1.68), frequent exposure to secondhand smoke (OR = 1.79), hospitalization ≥ 1 time in half a year (OR = 2.13), and history of allergic rhinitis (OR = 2.14) were risk factors for pneumonia in children. Wearing masks when outdoors (OR = 0.89) and engaging in regular physical activity (OR = 0.65) were protective factors. The pneumonia detection rate in children showed a decreasing trend during the COVID-19 pandemic in 2020 but an increasing trend after the pandemic in 2021. Premature birth, exposure to secondhand smoke, frequent hospitalization, and allergic rhinitis were risk factors for pneumonia in children. Wearing masks when outdoors and exercising may reduce the risk of pneumonia in children.

## 1. Introduction

On March 11, 2020, the World Health Organization declared the outbreak of novel coronavirus disease 2019 (COVID-19) a pandemic. Almost all the countries and regions were affected.^[1]^ Measures were taken to reduce infection rates, including social distancing, home isolation, and closing of shops and schools.^[2]^ However, these measures not only led to psychological problems (anxiety, loneliness, depression, etc)^[3]^ but also affected the physical health and nutritional status of people.^[4]^ Although children are less likely to be infected with COVID-19, it is important to recognize its potential harm of COVID-19. Therefore, this study aimed to analyze the time trends of childhood pneumonia before and after the COVID-19 pandemic in Laiwu, China, and explore the related risk factors to provide a reference for the prevention, control, and treatment of childhood pneumonia.^[5–8]^

## 2. Methods

### 2.1. Patients

Imaging examinations of children with pneumonia were performed using the GE-Optima multi-slice spiral CT and Philips Dura Diagnost digital radiography system. The scanning range was from the entrance of the chest to the base of the lungs. All CT data were reconstructed using lung and soft tissue windows with a slice thickness of 1.25 mm. In the examination report, 2 imaging physicians (attending physicians) analyzed the images and made the diagnosis of pneumonia or lung infection.^[9]^ According to the diagnostic criteria of the Guidelines for the Management of Community-Acquired Pneumonia in Children (revised 2013), the imaging results were combined with clinical symptoms and laboratory examinations to confirm the diagnosis of pneumonia.

The selection of risk factors in this study refers to the research methods of scholars at home and abroad^[10,11]^ and was partially revised according to the specific situation of the Laiwu area. The survey included the following 5 parts: Demographic characteristics, including sex and age; Feeding, whether the child is premature and breastfed; The child’s health, including whether the child has pollen allergy and the number of hospitalizations in the past 6 months; The children’s living environment, including whether pets are kept at home, whether the home has been decorated in the past year, and whether they are often exposed to secondhand smoke; and Lifestyle habits, including whether children wear masks when are outdoors and whether they often exercise. Before the start of the survey, the relevant personnel in our research group received uniform professional training, and the questionnaire was completed with the help of the child’s parents or other guardians. All questionnaires were checked by more than 2 trained members of the research group, and those with incomplete content were deleted.

### 2.2. Statistical analysis

We used Excel 2019 to create the case database and imported the data into SPSS26.0 software (IBM Corp., Armonk, NY) for data analysis. We compared the age variables using the *t* test and performed Pearson chi-square test for univariate analysis of other factors. We then used the Generalized Estimating Equations (GEE) model to analyze the pneumonia detection rate over time, adjusting for potential confounders. We selected the best GEE model based on the quasi-likelihood under the independence model criterion and used the Wald test to examine the significance of the model coefficients. We also included the factors with statistical significance in the multivariate logistic regression model to identify the risk and protective factors of childhood pneumonia. We used a 2-sided test with α = 0.05 as the significance level and considered *P* < .05 as statistically significant.

## 3. Results

### 3.1. General information

A total of 11,716 children, including 6101 boys (51.5%) and 5737 girls (48.5%), were enrolled. The minimum age was 3 years, and the maximum age was 12 years. The mean age was (5.14 ± 2.24) years. The detection rates of pneumonia were 41.5% (599/1444) in 2018, 39.5% (1738/4405) in 2019, 38.1% (1385/3634) in 2020, and 40.7% (1579/3865) in 2021. (Table 1)

**Table 1:** Statistical table of pneumonia detection in children in Laiwu, China, from 2018 to 2021.

Year	Total number of patients	Number of patients with pneumonia	Pneumonia detection rate (%)	χ^2^	*P* value
2018	1444	599	41.5	4.08	.25
2019	2289	905	39.5		
2020	1152	439	38.1		
2021	6831	2778	40.7		

The pneumonia detection rate was calculated as the number of patients with pneumonia divided by the total number of patients in each year. We used the Pearson chi-square test to compare the pneumonia detection rate across the four years. *P* < .05 was considered statistically significant.

### 3.2. Time trends of pneumonia detection rate

The GEE model showed that the pneumonia detection rate decreased by 3.4% in 2020 compared with that in 2018 (β=−0.034, *P* < .001) but increased by 2.6% in 2021 compared with that in 2020 (β = 0.026, *P* < .001). The pneumonia detection rate showed a decreasing trend in 2020 during the COVID-19 pandemic but an increasing trend in 2021 after the pandemic. (Table 2)

**Table 2:** Comparison of general conditions between the pneumonia and non-pneumonia groups (n = 11 716).

Factors	Pneumonia group	Non-Pneumonia group	t/χ^2^	*P* value
	(n = 4721)	(n = 6995)		
Age (yr)	5.08 ± 2.08	5.02 ± 2.04	1.57	.12
Sex				
Male	2432 (40.2%)	3619 (59.8%)		
Female	2289 (40.4%)	3376 (59.6%)	0.06	.81
Premature birth				
Yes	458 (52.5%)	414 (47.5%)		
No	4263 (39.3%)	6581 (60.7%)	58.56	<.01*
Breastfeeding				
Yes	2909 (40.8%)	4214 (59.2%)		
No	1812 (39.5%)	2781 (60.5%)	2.24	.14
Home pet ownership				
Yes	671 (40.1%)	1002 (59.9%)		
No	4050 (40.3%)	5993 (59.7%)	0.03	.87
Secondhand smoke exposure				
Yes	2538 (48.5%)	2690 (51.5%)		
No	2183 (33.6%)	4305 (66.4%)	267.15	<.01*
Hospitalized in the last 6 mo				
<1	4212 (38.8%)	6644 (61.2%)		
≥1	509 (59.2%)	351 (40.8%)	137.67	<.01*
Home renovation in the last yr				
Yes	677 (39.5%)	1036 (60.5%)		
No	4044 (40.4%)	5959 (59.6%)	0.50	.48
Pollen allergy				
Yes	671 (57.2%)	502 (42.8%)		
No	4050 (38.4%)	6493 (61.6%)	154.90	<.01*
Wearing masks when going out				
Yes	3137 (39.3%5)	4838 (60.7%)		
No	1584 (42.3%/)	2157 (57.7%)	9.57	<.01*
Physical exercise				
Yes	2351 (35.6%)	4256 (64.4%)		
No	2370 (46.4%)	2739 (53.6%)	139.82	<.01*

We compared the age variables using the t-test and performed Pearson chi-square test for univariate analysis of other factors in this table. *P* < .05 was considered statistically significant.

* indicates *P* < .05.

### 3.3. Risk factors for pneumonia in children

The results of multivariate logistic regression analysis showed that preterm birth (odds ratio [OR] = 1.68, 95% confidence interval [CI]: 1.46–1.94), frequent exposure to secondhand smoke (OR = 1.79, 95% CI: 1.66–1.93), hospitalization ≥ 1 time in half a year (OR = 2.13, 95% CI: 1.84–2.46), and history of allergic rhinitis (OR = 2.14, 95% CI: 1.89–2.43) were risk factors for childhood pneumonia. Wearing masks when outdoors (OR = 0.89, 95% CI: 0.83–0.97) and regular physical activity (OR = 0.65, 95% CI: 0.60–0.70) were protective factors. (Table 3).

**Table 3:** Multivariate logistic regression analysis of factors influencing pneumonia.

Factors	Reference group	β	S	Wald χ^2^	*P* value	OR	95% CI
Premature birth	Yes	0.52	0.07	51.14	.00	1.68	1.46–1.94
Secondhand smoke exposure	Yes	0.58	0.04	221.80	.00	1.79	1.66–1.93
Hospitalized in the last 6 months ≥ 1 time	Yes	0.76	0.07	103.72	.00	2.13	1.84–2.46
Home renovation in the last year	Yes	0.05	0.06	0.91	.34	1.05	0.95–1.18
Pollen allergy	Yes	0.76	0.06	138.60	.00	2.14	1.89–2.43
Wearing masks when going out	Yes	−0.11	0.04	7.28	.03	0.89	0.83–0.97
Physical exercise	Yes	−0.43	0.04	119.75	.00	0.65	0.60–0.70

We used the multivariate logistic regression model to analyze the factors influencing pneumonia in children, after adjusting for other factors in this table. P < .05 was considered statistically significant.

CI = confidence interval, OR = odds ratio.

## 4. Discussion

In this study, the overall pneumonia detection rate in children was 40.3% from 2018 to 2021 in Laiwu, China, which decreased during the COVID-19 pandemic in 2020 but increased after the pandemic in 2021. This may be related to the strict implementation of isolation measures such as wearing masks, social distancing, and avoiding going out at the beginning of the pandemic, which reduced the spread of pneumonia-causing pathogens and pneumonia detection rate in children.^[10]^ However, with the easing of the pandemic and relaxation of relevant isolation measures, people’s lifestyles have changed, such as less wearing of masks in public places and more outdoor activities and gatherings.

The results of multivariate logistic regression analysis showed that preterm birth, exposure to secondhand smoke, frequent hospitalization, and allergic rhinitis were risk factors for pneumonia in children. During the COVID-19 pandemic, some restrictive measures effectively contained the spread of COVID-19 but indirectly led to changes in children’s diets and reduced outdoor physical activity,^[12,13]^ which may explain the surge in childhood pneumonia cases in 2021. The COVID-19 pandemic is ongoing worldwide, and many countries and regions are suffering from the pandemic. Therefore, relevant departments should formulate targeted guidelines and policies for children and ensure the health of children during public health emergencies leading to lockdowns.^[14]^ In addition, children should develop the habit of wearing masks when going out and actively participating in physical activity.^[15]^

Pneumonia is a major cause of death in children that poses a serious health threat and also consumes large amounts of medical resources during treatment. Therefore, it is necessary to strengthen research on the epidemiological trends and etiology of pneumonia in children.

The limitations of this study are that it was conducted in a city that had been in a low-risk state for a long time, and there were no data on pneumonia in children from high-risk areas of COVID-19 for comparison. In addition, the diagnostic criteria for pneumonia in this study were based on imaging findings, and the detection rate would be higher than that of laboratory test results, because patients with mild pneumonia may not undergo imaging tests;^[16]^ these limitations may lead to certain biases in the results.^[17]^ For a more comprehensive and accurate understanding of the etiology and epidemiological trends of pneumonia in children, larger sample data and multicenter collaborative studies are needed.^[18,19]^

## 5. Conclusion

The pneumonia detection rate in children showed a decreasing trend during the COVID-19 pandemic in 2020 but an increasing trend after the pandemic in 2021 in Laiwu, China. Premature birth, exposure to secondhand smoke, frequent hospitalization, and allergic rhinitis were risk factors for childhood pneumonia. Wearing masks when going out and exercising can reduce the risk of pneumonia in children. Relevant departments should pay attention to the health status of children during public health emergencies and encourage them to increase their personal protective measures such as wearing masks and participating in outdoor activities.

## Acknowledgements

We would like to thank all the study participants and their parents/guardians.

## Author contributions

**Conceptualization:** Jimei Yang, Ruiliang Lv, Mingzhu Zhang.

**Data curation:** Ruiliang Lv, Chunfeng Zheng, Yan Gu.

**Formal analysis:** Mingzhu Zhang.

**Methodology:** Junqing Li, Xiang Wang, Chunfeng Zheng.

**Project administration:** Chunfeng Zheng.

**Resources:** Jimei Yang, Ruiliang Lv, Yan Gu.

**Software:** Xiang Wang.

**Writing – original draft:** Jimei Yang, Junqing Li.

**Writing – review & editing:** Junqing Li.
